# A cancer-associated mutation inactivates a region of the high-mobility group protein HMG20b essential for cytokinesis

**DOI:** 10.4161/15384101.2014.942204

**Published:** 2014-10-30

**Authors:** MiYoung Lee, Ashok R Venkitaraman

**Affiliations:** University of Cambridge; Medical Research Council Cancer Unit; Hutchison/MRC Research Center; Cambridge, UK

**Keywords:** BRCA2, cytokinesis, HMG20b, somatic mutations in cancer, tumor suppression

## Abstract

Defects in the completion of cell division by cytokinesis have long been proposed to foster carcinogenesis by engendering chromosome instability, but few tumor suppressor mechanisms controlling this process have so far been identified. Here, we identify a carboxyl (C)-terminal region of the high-mobility group protein HMG20b that is essential for cytokinesis, and report that it is inactivated by a cancer-associated mutation. We find that a C-terminal region of HMG20b spanning residues 173–317 is necessary and sufficient not only for its localization to cytokinetic structures, but also for its interaction with the tumor suppressor BRCA2, implicated in the abscission step of cytokinesis. Indeed, expression of this C-terminal HMG20b region suffices to restore cytokinesis in HMG20b-depleted cells. The non-conservative substitution of HMG20b residue Ala247 with Pro, reported in human lung cancer, disrupts these activities of HMG20b, impairing cytokinesis in a *trans*-dominant manner. Our findings provide fresh insight into the mechanism by which the HMG20b-BRCA2 complex controls mitotic cell division, and implicate heterozygous HMG20b mutations affecting cytokinesis regulation in the genesis of human cancers.

## Abbreviations

A247P: alanine to proline substitution at position 247
BRCA2: breast cancer 2, early onset
COSMIC: Catalogue of Somatic Mutations in Cancer
Dox: doxycycline
EGFP: enhanced green fluorescent protein
ESCRT: endosomal sorting complex required for transport
GST: glutathione S-transferase
HMG: high mobility group
KLCC: kinesin-like coiled coil
Luc: luciferase
Tet: Tetracycline
UTR: untranslated regions
V312G: valine to glycine substitution at position 312

## Introduction

A long-standing hypothesis concerning human carcinogenesis is that the failure of cell division, which generates polyploid cells containing an abnormally high number of chromosomes, can provoke the formation of aneuploid tumors.^1-4^ Normally, mitotic cell division culminates in cytokinesis, during which the cellular and genetic complement of a cell is divided equally into 2 daughters (reviewed in ^5,6^). Cytokinesis begins when a cortical, actomyosin-based contractile ring forms at the equator of the dividing cell following the metaphase-to-anaphase transition, which subsequently constricts during anaphase to form a structure called the cleavage furrow. The cleavage furrow eventually compacts the central region of the mitotic spindle into the midbody, which forms part of a narrow intracellular bridge that is cleaved during abscission to complete the physical separation of the daughter cells. Surprisingly, whereas there is growing insight into the molecules and mechanisms involved in contractile ring formation, cell cleavage and abscission during mitosis, few of these mechanisms have so far been connected to human carcinogenesis.^7,8^

Here, we report evidence that implicates HMG20b, a ubiquitously expressed protein of uncertain function, in tumor suppression through an essential role performed by its carboxyl (C)-terminal region in cytokinesis. HMG20b was first isolated as a member of the high-mobility group (HMG) of non-sequence-specific DNA binding proteins,^9^ contains an N-terminal HMG domain and a C-terminal coiled-coil region. Little is known of HMG20b's biological functions. It has previously been reported to decorate condensed mitotic chromosomes and regulate cell cycle progression from G2 into mitosis,^10^ to repress the expression of neuronal genes through participation in a histone-deactylase complex,^11,12^ and to bind to BRCA2, a hereditary breast and ovarian cancer suppressor protein,^10,13^ to regulate cell division by cytokinesis.^13^ We have investigated the mechanism by which the BRCA2-HMG20b complex regulates cell division, and demonstrate here that the C-terminal region of HMG20b is necessary and sufficient for complex formation with BRCA2, for localization to cytokinetic structures, and for the restoration of cytokinesis in HMG20b-depleted cells. Interestingly, a non-conservative substitution of Ala247 to Pro within this critical HMG20b region, recently reported in human lung cancer, suffices to inactivate these activities of HMG20b. Collectively, our findings define a mechanism that regulates normal cell division by cytokinesis, and its disruption by a cancer-associated mutation.

## Results

### The C-terminal region of HMG20b mediates binding to the BRC5 motif in BRCA2

**Figure 1.**
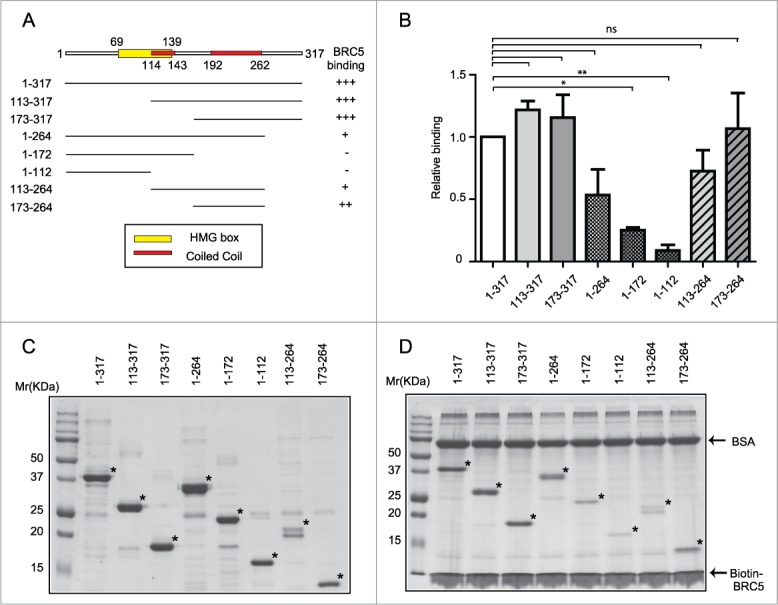
The C-terminal region of HMG20b mediates complex formation with BRCA2. (**A**) Domain structure of HMG20b and design of N-terminal and C-terminal HMG20b fragments. The boundaries are shown as amino acid residue numbers. Binding affinity for BRC5 is shown next to each fragment. (**B**) Relative binding affinity of each HMG20b fragment for BRC5 repeat. Binding affinity was calculated by dividing band intensities of bound fractions by those of input. Relative binding affinity of each fragment compared to the full-length protein is shown as means ± s.e.m. from 3 independent experiments. Each HMG20b fragment is compared with the full-length HMG20b by Dunnett's multiple comparison test (**: 0.001 < *P* < 0.01, *: 0.01 < *P* < 0.05, ns: *P* > 0.05). (**C**) Coomassie brilliant blue staining of gel showing purified HMG20b fragments (marked with an asterisk). (**D**) C-terminal region of HMG20b mediates binding to BRC5 motif. Coomassie brilliant blue staining of gel from Streptavidin pull-down assay. HMG20b fragments bound to Biotin-BRC5 peptide are marked with an asterisk. BSA included in excess amount in the binding buffer appears after staining of the gel.

To investigate further how complex formation between HMG20b and BRCA2 regulates cytokinesis, we defined the region within HMG20b required for this interaction. Human HMG20b contains at its amino (N)-terminus at amino acids (aa) 69–139 (**Fig. 1A**) a high mobility group (HMG) domain, implicated in non-sequence specific DNA binding. *In silico* structural analysis also predicted the presence of a kinesin-like coiled coil (KLCC) domain, consisting of a heptamer repeat similar to a leucine zipper, spanning aa238–305.^10,14^ However, more recently developed coiled-coil predicting programs, Pair Coil 2 and COILS^15,16^ predict a more extensive coiled-coil domain extending across aa114–143 and 192–262 (**Fig. 1A**), forming a bundle of α-helices that may provide interfaces for the dynamic assembly of protein complexes.^17^ We therefore designed a series of truncated fragments of HMG20b protein, including or excluding the predicted coiled-coil domain (**Fig. 1A**) and encoding C-terminal 6xHis tags, to test the importance of the domain in complex formation with BRCA2. These His-tagged HMG2b fragments were expressed in *E. coli* and purified using a nickel-charged affinity resin (**Fig. 1C**). We have previously shown^13^ that HMG20b binds to the BRC5 repeat motif spanning residues 1661–1695 within human BRCA2. Notably, 2 C-terminal HMG20b fragments (spanning aa113–317 and aa173–317) exhibited significant binding to a biotinylated peptide encoding the BRC5 motif (**Fig. 1B** and **D**). These findings define the HMG20b region spanning residues 173–317 as the minimal region required for complex formation with the BRC5 repeat of BRCA2.

We further dissected this minimal region aa173-317 (**Fig. S1**) by serial truncation from the C-terminus. Whereas the aa173–276 fragment retained BRC5 binding, further truncation (fragment aa173–264) caused a reduction in the binding activity. Moreover, the short fragment at the extreme C-terminus of HMG20b (aa 265–317) did not bind to the BRC5 repeat (**Fig. S1B** and **D**).

Collectively, our results identify the region between HMG20b residues 173–276 as the minimal requirement for efficient binding to the BRC5 repeat of BRCA2. Although the predicted coiled-coil domain (aa192–262) falls within this minimal region, a shorter fragment aa173–264, which also contains the coiled-coil domain, exhibits reduced binding activity. Also, the upstream coiled-coil region (114–143) was not required for the binding to the BRC5 repeat. These results indicate that the coiled-coil structure alone is insufficient for HMG20b-BRCA2 binding, and that additional features within HMG20b also cooperate.

### The C-terminal region of HMG20b mediates midbody localization

**Figure 2.**
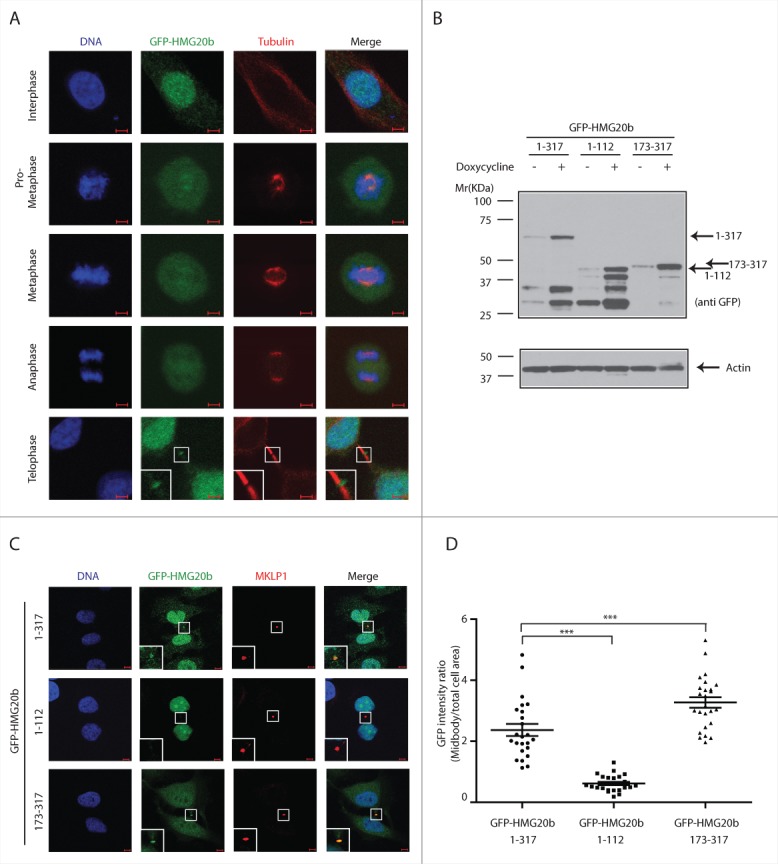
The C-terminal region of HMG20b mediates localization to midbody. (**A**) Localization of HMG20b during cell cycle. Confocal images of HeLa cells expressing GFP-HMG20b are shown for each cell cycle phase. Enlarged image of midbody is shown as an inset . Scale bar is 5 μm. (**B**) Western blot showing inducible expression of GFP-HMG20b fragments. β-Actin was probed as a loading control. (**C**) Confocal images of HeLa cells expressing GFP-HMG20b fragments (green). MKLP1(red) was immunostained as a midbody marker. Enlarged images of midbody are shown as insets. Scale bar is 5 μm. (**D**) GFP intensity at midbody normalized to the intensity within total cell area for each GFP-HMG20b fragments. A representative scatter dot plot from 3 independent experiments is shown with mean ± s.e.m. Twenty-five cells were analyzed in each experiment. Statistical significance was confirmed for the indicated pair-wise comparisons using Dunnett's multiple comparison test (***: *P* < 0.001).

BRCA2 is recruited to the midbody by the actin-binding protein Filamin A, which is required for subsequent recruitment of the endosomal sorting complex required for transport (ESCRT) components, Alix, and Tsg101 to the midbody and formation of CEP55-Alix and CEP55-Tsg101 complexes during abscission.^18^ Whether HMG20b also localizes to the midbody has not yet been ascertained. To address this issue, we created HeLa cell lines which express Tetracycline (Tet)-inducible constructs encoding HMG20b tagged at its N-terminus with enhanced green fluorescent protein (GFP). We find that GFP-HMG20b is nuclear during interphase. Upon entry into mitosis, HMG20b redistributes to the whole cell area with slight concentration on the mitotic spindle (**Fig. 2A**). However, it is excluded from condensed chromosomes (**Fig. 2A**, **Fig. S2**), in contrast to a previous report by Marmorstein et al.^10^ HMG20b is finally recruited to the cytokinetic midbody during telophase (**Fig. 2A**), which is consistent with the proposed function for the HMG20b-BRCA2 complex in abscission.^13^

**Figure 3.**
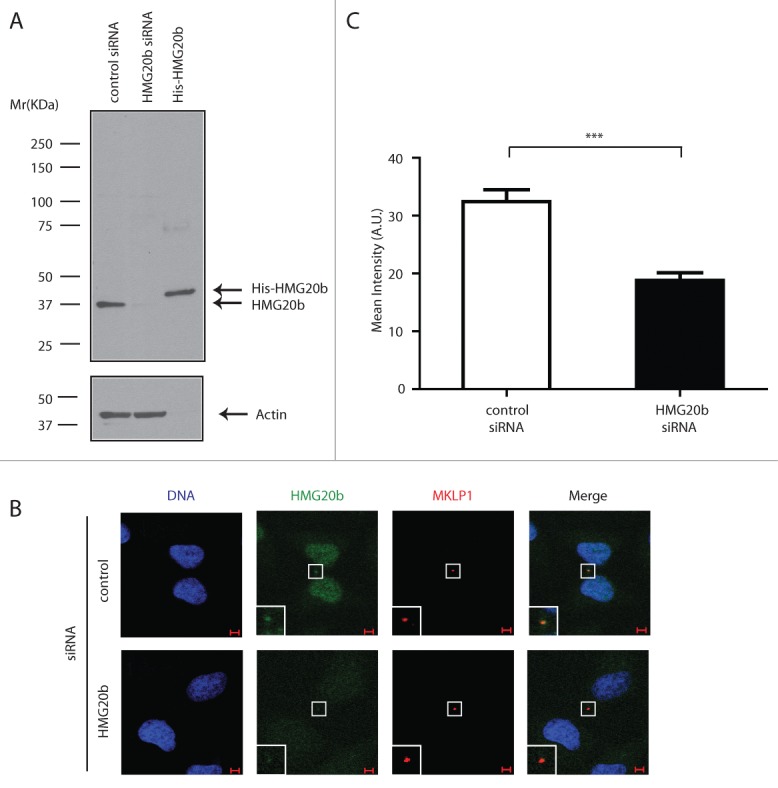
Endogenous HMG20b localizes to the cytokinetic midbody. (**A**) Western blot analysis of control and HMG20b siRNA-treated HeLa cell extracts using HMG20b monoclonal antibody 1F6. Purified His-HMG20b protein was used as a positive control and β-Actin was probed as a loading control. (**B**) Confocal images of control and HMG20b siRNA-treated HeLa cells stained with anti-HMG20b monoclonal antibody 1F6 (green). MKLP1 (red) was co-stained as a midbody marker. Enlarged images of midbody are shown as insets. Scale bar is 5 μm. (**C**) Mean intensity of HMG20b staining at midbody. Results from 50 cells per sample are shown with mean ± s.e.m. (***: *P* < 0.001, unpaired t test)

HMG20b localization to the cytokinetic midbody during telophase was also verified by staining for the endogenously expressed HMG20b protein. The monoclonal antibody clone IF6 specifically recognizes a single band corresponding to the expected molecular weight of HMG20b in Western blotting, which is depleted following exposure to HMG20b siRNA (**Fig. 3A**). Indeed, IF6 staining decorates the midbody, and is specifically reduced following HMG20b depletion (**Fig. 3B**, **C**). These results provide evidence that endogenous HMG20b, as well as GFP-HMG20b, localize to the cytokinetic midbody.

Interestingly, GFP-HMG20b co-staining with midbody markers such as MKLP1 and Cep55 not only confirms midbody localization, but also suggests that Cep55 and HMG20b occupy distinct topologic domains within the structure. Whereas the spatial overlap between GFP-HMG20b and MKLP staining is virtually complete (**Fig. S3A**), Cep55 and GFP-HMG20b only partly coincide, suggesting that they flank each other rather than occupy the same location^19^ (**Fig. S3B**).

Notably, a GFP-tagged form of the C-terminal fragment of HMG20b spanning residues 173–317, which includes the BRCA2-binding region, is efficiently localized to the cytokinetic midbody (**Fig. 2C**). Its accumulation at this site is comparable to that of full-length GFP-HMG20b expressed at a similar level (**Fig. 2B** and **D**). In contrast, an N-terminal fragment (GFP-HMG20b 1-112) did not exhibit midbody localization. Interestingly, the C-terminal fragment evenly distributes between the nucleus and cytoplasm, whereas the full-length protein and the N-terminal fragment are predominantly nuclear, which is consistent with the presence of a putative NLS (nuclear localization signal) at the N-terminal region (aa 52–66) of the protein.^20^ Thus, our results suggest that a C-terminal fragment of HMG20b required for complex formation with BRCA2 is sufficient for its localization to cytokinetic structures.

### The C-terminal region is necessary and sufficient for an essential role of HMG20b in cytokinesis

We have previously shown that depletion of HMG20b leads to delayed or failed cytokinesis.^13^ To test whether the C-terminal region of the protein is involved in this role, we established a series of HeLa cell clones stably transfected with Tet-inducible cDNA constructs encoding different regions of HMG20b fused at their N-termini to a FLAG-epitope tag (**Fig. S4**). The selected HeLa cell clones express comparable levels of the different HMG20b fragments after treatment with the inducer, Doxycycline (**Fig. S6**). Since the constructs encoding these proteins lack the untranslated regions (UTR) present in the mRNA for endogenous HMG20b, we used 3 different siRNA duplexes (D4, D5 and Q1, **Fig. S5**) targeting the 3’ UTR of HMG20b mRNA to deplete endogenous HMG20b. All 3 siRNA duplexes, which were designed using 2 different algorithms to minimize seed sequence homologies, showed a similar efficiency in inducing cytokinesis failure marked by the appearance of multi-nucleate cells (**Fig. S5**). We therefore selected the siRNA duplex Q1 for use in subsequent experiments, and confirmed that it could efficiently deplete endogenous HMG20b protein without affecting the expression of the stably transfected constructs (**Fig. S6**).

**Figure 4.**
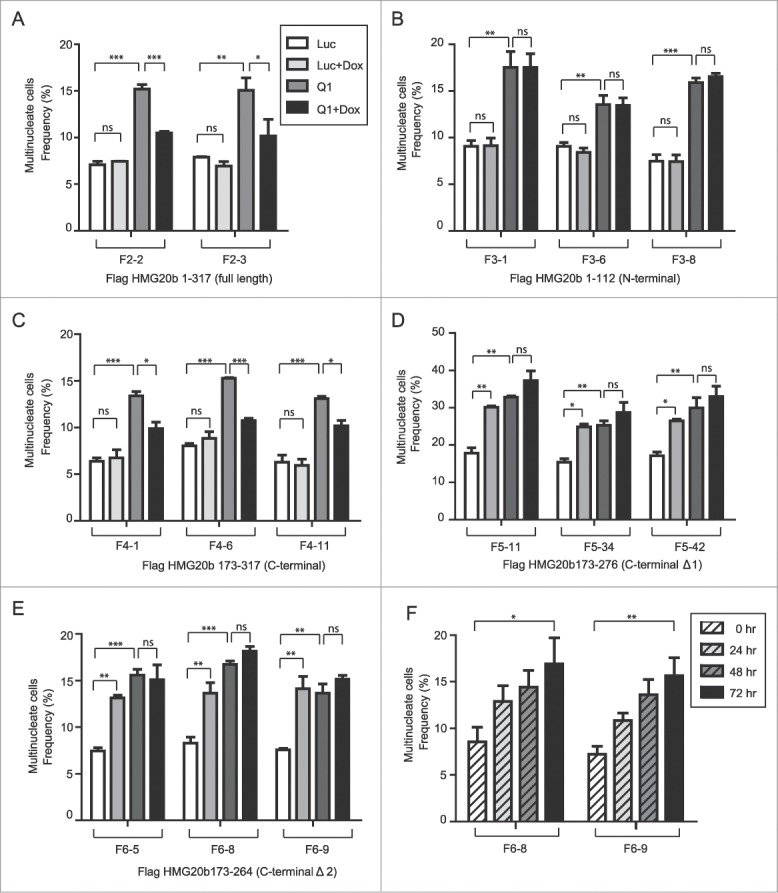
The C-terminal region is necessary and sufficient for an essential role of HMG20b in cytokinesis. (**A**) Frequency of multinucleate cells was analyzed in 2 independent HeLa cell clones (F2-2 and F2-3) expressing full-length Flag-HMG20b following treatment with HMG20b siRNA (Q1) and/or Doxycycline (Dox). Means ± s.e.m. from 3 independent experiments are shown and >500 cells were analyzed per each sample. Statistical significance was confirmed for the indicated pairwise comparisons using Bonferroni's multiple comparison test (***: *P* < 0.001, **: 0.001 < *P* < 0.01, *: 0.01 < *P* < 0.05, ns: *P* > 0.05). The same treatments (siRNA ± Dox) and statistical analysis were applied to Figure 4 (**B**), (**C**), (**D**) and (**E**). (**B**) Three independent clones (F3-1, F3-6, and F3-8) expressing N-terminal (1-112) HMG20b fragment were analyzed for multinucleation frequency. (**C**) Three independent clones (F4-1, F4-6, and F4-11) expressing C-terminal (173–317) HMG20b fragments were analyzed for multinucleation frequency. (**D**) Three independent clones (F5-11, F5-34, and F5-42) expressing truncated C-terminal fragment Δ1 (173–276) were analyzed for multinucleation frequency. (**E**) Three independent clones (F6-5, F6-8, and F6-9) expressing truncated C-terminal fragment Δ2 (173–264) were analyzed for multinucleation frequency. (**F**) Clones F6-8 and F6-9 were treated with Doxycycline and analyzed for multinucleation frequency every 24 hours for 3 d. >500 cells were analyzed for each sample. Means ± s.e.m. from 3 independent experiments are shown (test for linear trend, **: 0.001 < *P* < 0.01, *: 0.01 < *P* < 0.05).

Notably, doxycyline (Dox)-induced expression of full-length FLAG-HMG20b protein in 2 independently-derived HeLa cell clones F2-2 or F2-3 (**Fig. S6A**) reduced in a statistically significant manner the occurrence of multi-nucleated cells marking cytokinesis failure after endogenous HMG20b depletion (**Fig. 4A**, **Fig. S7**). This result further validates that HMG20b performs an essential role during normal cytokinesis.^13^ However, expression of the N-terminal fragment FLAG-HMG20b 1–112 failed to restore cytokinesis under similar conditions in 3 independently-derived cell clones F3-1, F3-6 or F3-8 (**Fig. 4B**, **Fig. S6B, Fig. S7**).

By contrast, expression of the C-terminal fragment FLAG-HMG20b 173–317 that contains the region required for BRCA2-HMG20b complex formation was sufficient to restore cytokinesis following endogenous HMG20b depletion in 3 independently-derived cell clones F4-1, F4-6 or F4-11 (**Fig. 4C**, **Fig. S6C, Fig. S7**). Notably, this C-terminal HMG20b fragment reduced the frequency of multinucleate cells marking failed cytokinesis to the same extent as full-length HMG20b (compare **Fig. 4A** with **4C**), suggesting that it is necessary and sufficient to supplant an essential role of HMG20b during cytokinesis.

We therefore further dissected the functional role of residues within this region. The C-terminal HMG20b fragments spanning aa 173–276 (C-terminal Δ1) and 173–264 (C-terminal Δ2) not only failed to restore cytokinesis in HMG20b-depleted cells (**Fig. 4D** and **E**, **Fig. S6D** and **E, Fig. S7**), but instead appeared to inhibit cytokinesis in cells treated with a control siRNA against luciferase (**Fig. 4D** and **E**, compare Luc with Luc+Dox). This was confirmed in a time-course experiment where expression of FLAG-HMG20b 173–264 increased the frequency of multinucleate cells marking cytokinesis failure in a time-dependent manner (**Fig. 4F**). Remarkably, the C-terminal HMG20b fragment spanning aa 173–276 (C-terminal Δ1) has similar BRC5 binding activity to the fragment 173–317. However, we find that it fails to restore cytokinesis after depletion of endogenous HMG20b protein, suggesting that additional C-terminal sequences are required. Thus collectively, our findings define a C-terminal region in HMG20b required for complex formation with BRCA2 that is necessary and sufficient for cytokinesis, and show that omission of even a small stretch of 41 residues at the very C-terminus of the BRCA2-binding region is enough to impair this function.

### A cancer-associated mutation in the C-terminal region of HMG20b disrupts cytokinesis in a dominant manner

Mutations in the *HMG20B* gene recently detected through next-generation sequencing of human cancer samples (Catalogue of Somatic Mutations in Cancer (COSMIC), http://www.sanger.ac.uk/cosmic^21^) are shown in **Table S1**. These mutations occur in several different forms of human epithelial cancer, including lung carcinomas, and affect a single *HMG20B* allele, prompting questions as to whether they affect HMG20b function despite the presence of a normal allele.

**Figure 5.**
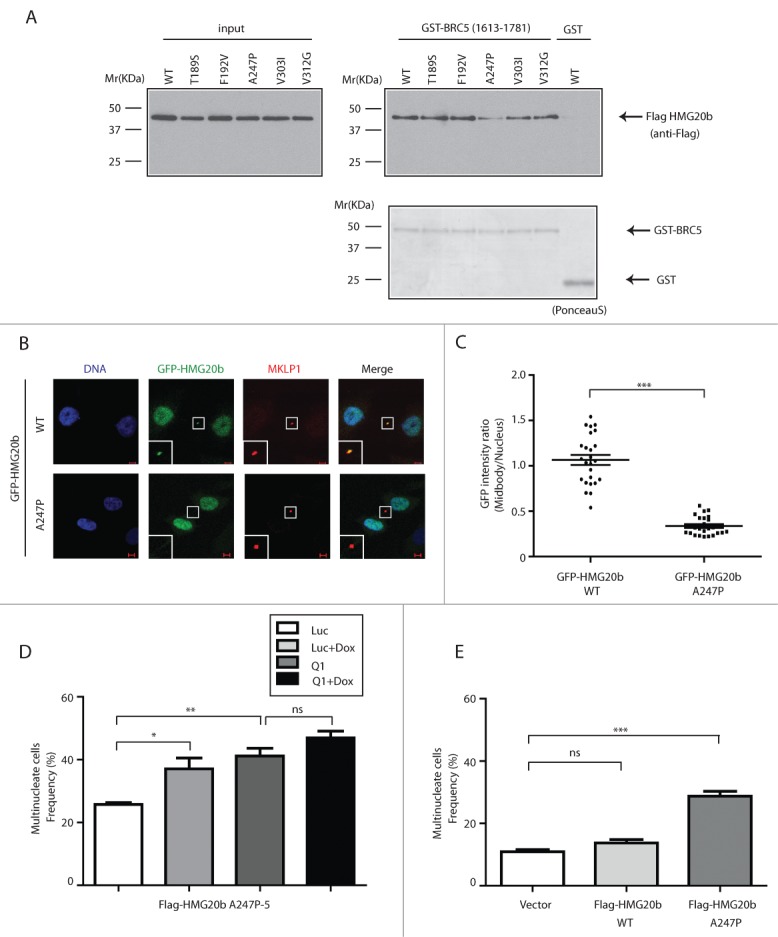
A cancer-associated mutation in the C-terminal region of HMG20b disrupts cytokinesis in a dominant manner. (**A**) GST-pull down assay. Flag-HMG20b mutants expressed in 293T cells were incubated with GST-BRCA2 fragment containing BRC5. Bound proteins were detected by western blotting using anti Flag antibody. Ponceau S- stained membrane is included in the lower panel to show that equal amount of GST fusion proteins was used for each sample. (**B**) Confocal images of HeLa cells expressing wild type or A247P mutant of GFP-HMG20b (green). MKLP1 (red) was immunostained as a midbody marker. Enlarged images of midbody are shown as insets. Scale bar is 5 μm. (**C**) Localization of wild type and A247P mutant of GFP-HMG20b at midbody. A representative scatter dot plot of GFP intensity ratio from 3 independent experiments is shown with mean ± s.e.m. Twenty-five cells were analyzed in each experiment (***: *P* < 0.001, unpaired t test). (**D**) Clone (A247P-5) expressing Flag-HMG20b with A247P mutation was analyzed for multinucleation frequency after HMG20b siRNA (Q1) and/or Doxycycline (Dox) treatment. Means ± s.e.m. from 3 experiments are shown and >500 cells were analyzed per each sample. Statistical significance was confirmed for the indicated pairwise comparisons using Bonferroni's multiple comparison test (**: 0.001 < *P* < 0.01, *: 0.01 < *P* < 0.05, ns: *P* > 0.05). (**E**) Multinucleation frequency measured at 72 hours after HeLa cells were transiently transfected with Flag-HMG20b constructs . Means ± s.e.m. from 4 independent experiments (2 experiments for the vector control) are shown. Transfected cells were identified by anti-Flag immunostaining and >200 transfected cells were analyzed per each sample. Wild type and A247P mutant of HMG20b are compared with the vector control by Dunnett's multiple comparison test (***: *P* < 0.001, ns: *P* > 0.05).

We tested the functional impact of several of these mutations, including T189S, F192V, A247P and V303I and V312G on the ability of mutant full-length FLAG-HMG20b to bind to a glutathione S-transferase (GST)-tagged fragment of human BRCA2 aa1613–1781 that contains the BRC5 motif (**Fig. 5A**; **Fig. S8A**). Notably, only one of these mutations (a non-conservative substitution of Ala247 with Pro (A247P)) reduced binding to the GST-BRCA2 fragment (**Fig. 5A**; **Fig. S8A**). Moreover, the A247P mutation also interferes with the localization of full-length GFP-HMG20b A247P protein to the cytokinetic midbody (**Fig. 5B and C**). These findings suggest that the cancer-associated A247P mutation in HMG20b compromises its normal function in BRCA2 binding and cytokinesis.

Interestingly, conditional expression of FLAG-HMG20b A247P in HeLa cells proved technically challenging. Few stably transfected HeLa clones could be isolated in this way, and those that were, exhibited background levels of multinucleation even when expression of the mutant protein was relatively low before induction with Doxycycline, suggesting that the mutation had a dominant-negative effect on cytokinesis. Indeed, as exemplified in the clone A247P-5, Dox-mediated induction of FLAG-HMG20b A247P or depletion of endogenous HMG20b caused a marked increase in the frequency of multinucleate cells marking cytokinesis failure, which was further accentuated by combining these manipulations (**Fig. 5D**; **Fig. S8B**). Moreover, even transient expression of FLAG-HMG20b A247P in cells expressing endogenous HMG20b sufficed to increase the frequency of multinucleate cells (**Fig. 5E**; **Fig. S8C**). Together, these results strongly suggest that the cancer-associated A247P mutation is not only functionally deleterious, but also that it can induce cytokinesis failure and aneuploidy in a dominant manner even when wild-type HMG20b is also present.

## Discussion

Our findings identify a C-terminal region of HMG20b that is necessary and sufficient not only for its localization to the cytokinetic midbody, but also for its interaction with the tumor suppressor BRCA2, implicated in the abscission step of cytokinesis. Indeed, both endogenous and GFP-tagged forms of HMG20b are recruited to the cytokinetic midbody consistent with participation in a BRCA2-HMG20b complex that regulates cell division. Although GFP-HMG20b co-localizes with midbody markers such as MKLP1 and Cep55, we observe differences in its staining pattern that suggest they occupy distinct topologic domains within the midbody structure. This observation warrants future investigation in the light of known mechanisms that regulate midbody assembly and function.^19^

We find that C-terminal HMG20b fragments spanning aa 173–276 and 173–264 (which lack a short stretch of residues at the very C-terminal region), as well as a cancer-associated mutation HMG20b mutant, A247P, not only fail to restore cytokinesis in HMG20b-depleted cells but also provoke defective cytokinesis even when endogenous wild-type HMG20b is present. This dominant negative effect cannot plausibly be exerted via competition with endogenous wild-type HMG20b for binding to BRCA2, since neither the aa 173–264 fragment nor the point-mutant form of HMG20b exhibit BRC5 binding. Therefore, we speculate that mutant HMG20b may interfere with the function of its wild-type counterpart through binding to a cytokinetic regulator other than BRCA2. Identification of such a putative mechanism warrants further investigation, but will require extensive biochemical studies beyond the scope of the current work.

In conclusion, our findings provide fresh insight into topical observations concerning the biological function of the BRCA2-HMG20b complex in the completion of mitotic cell division by cytokinesis.^13,18,22^ BRCA2 interacts with HMG20b via the evolutionarily conserved BRC5 motif,^13,23,24^ but with other cytokinetic mediators such as BCCIP,^22^ Filamin A, CEP55 and ESCRT components^18^ using distinct regions, suggesting that BRCA2 may serve as a scaffold for the dynamic assembly of a large, multi-protein complex at cytokinetic structures such as the midbody.

Importantly, we show that a mutant form of HMG20b detected in human epithelial cancers acts in a dominant manner to induce cytokinesis failure. Our results suggest that *HMG20B* may represent an unusual type of human tumor suppressor gene, whose heterozygous alteration by mis-sense mutations may suffice to promote carcinogenesis.^25^ Our work also serves to exemplify and substantiate the long-standing proposal that defects in the completion of cell division may foster chromosome instability and human carcinogenesis. These insights collectively raise numerous important issues warranting future investigation.

## Materials and Methods

### Protein expression and purification

Various fragments of human HMG20b cDNA (as outlined schematically in **Fig. 1A****; Fig. S1A**) were cloned into pET21a vector (MerckMillipore, 69740-3). HMG20b fragments with C-terminal His-tag were expressed in *E. coli* BL21 (DE3) (Bioline, BIO-85032) and purified using Ni-NTA agarose beads (Qiagen, 30210). GST-BRCA2 fragment (1613–1781) containing BRC5 repeat was cloned in pGEX-4T3 vector (GE Healthcare, 28-9545-52) and purified as described previously.^26^

### Streptavidin pull-down assay

Biotinylated BRC5 peptide (amino acid residues 1661–1695) was synthesized by Cambridge Research Biochemicals. For the pull-down assay, 1 nmol of biotinylated peptide bound to Dynabeads M-280 Streptavidin (Invitrogen, 11205D) was incubated with 100 pmol of purified HMG20b fragments in binding buffer (50mM Tris-HCl (pH 7.4), 150mM NaCl, 1% NP-40, 5mM EDTA, 10mM NaF, and complete protease inhibitor cocktail (Roche, 11873580001)) containing 0.1% BSA for 30 minutes at room temperature. The beads were washed with binding buffer to remove unbound proteins and the bound proteins were then detected by staining with Coomassie brilliant blue R-250 (BioRad, 161-0436) following electrophoresis using 12% Tris-Glycine gels.

### GST pull-down assay

GST-BRCA2 fragment bound to Glutathione Sepharose Beads (GE Healthcare, 17-0756-01) was incubated with 293T cell extracts expressing Flag-HMG20b in binding buffer (50mM Tris-HCl (pH 7.4), 300mM NaCl, 1% NP-40, 5mM EDTA, 10mM NaF, and complete protease inhibitor cocktail (Roche)) containing 0.1% BSA for 30 minutes at room temperature. The beads were washed extensively with the binding buffer and bound proteins were analyzed by western blotting with the indicated antibodies.

### siRNA sequences

Luciferase siRNA (control): 5'-CGUACGCGGAAUACUUCGA-3' (synthesized by Eurofins MWG Operon),

D4: 5'-GGACACAGGGCAGACGAAA-3' (D-020146-04, Thermo Scientific),

D5: 5'-CGCGAUACACCCAGAAGAA-3' (D-020146-05, Thermo Scientific),

Q1: 5'-CAGCATCCCTTTAGCTTTCAA-3' (SI00087633, Qiagen)

### Mutagenesis

HMG20b mutant constructs were made by PCR using AccuPrime Pfx DNA polymerase (Invitrogen, 12344-024). PCR products were digested with DpnI enzyme and were used to transform *E. coli* DH5α. The following primers were used for mutagenesis.

T189S:

Forward 5'CCACCTTCGATGTTCCCATCTTCAGTGAAGAGTTCTTGGACC3'

Reverse 5'GGTCCAAGAACTCTTCACTGAAGATGGGAACATCGAAGGTGG3'

F192V:

Forward 5'CCCATCTTCACTGAAGAGGTCTTGGACCAAAACAAAGCGCG3'

Reverse 5'CGCGCTTTGTTTTGGTCCAAGACCTCTTCAGTGAAGATGGG3'

A247P:

Forward 5'GAGGAGCGGAGGACGCTGCCGCTGCAGCAGCAGCTCCAG3'

Reverse 5'CTGGAGCTGCTGCTGCAGCGGCAGCGTCCTCCGCTCCTC3'

V303I:

Forward 5'GCACGAGAAGCTCATCATCCGCATCAAGGAAATCCTGGCC3'

Reverse 5'GGCCAGGATTTCCTTGATGCGGATGATGAGCTTCTCGTGC3'

V312G:

Forward 5'GGAAATCCTGGCCCAGGGCGCCAGCGAGCACCTG3'

Reverse 5'CAGGTGCTCGCTGGCGCCCTGGGCCAGGATTTCC3'

### Inducible expression of HMG20b fragments

A Tet-On inducible gene expression system (Clontech, 630922) was used to conditionally express HMG20b fragments. The indicated HMG20b fragments (as shown in **Fig. S4**) were first cloned into EcoRI and BamHI sites of p3xFLAG-CMV-10 vector (Sigma, E7658). The region containing Flag-tag and HMG20b fragments were amplified by PCR and cloned into BamHI and MluI sites of pTRE2pur vector. The constructs were used to transfect HeLa Tet-On advanced cell line (Clontech, 632110) and stable clones were selected with puromycin (1μg/ml). For expression of GFP-HMG20b fragments, respective HMG20b cDNA fragments were first cloned into EcoRI and BamHI sites of pEGFP-C1 vector (Clontech, 6084-1). The region containing GFP-tag and HMG20b fragments were amplified by PCR and cloned into BamHI and MluI sites of pTRE2pur vector as described above.

### siRNA transfection and induction of Flag-HMG20b fragment expression

Cells were seeded on 6-well plates in the medium with or without Doxycline (1 μg/ml) the day before the transfection with siRNAs (40 nM) using Lipofectamine 2000 reagent (Invitrogen, 11668-027). Cells were split into 35 mm culture dishes 24 hours post-transfection and phase-contrast images were taken at 72 hours post-transfection. +Dox samples were continuously incubated with Doxycycline during the experiment.

### Transient expression of Flag-HMG20b

Flag-HMG20b constructs were transfected into HeLa or 293T cells using jetPRIME transfection reagent (Polyplus transfection, 114-07) according to the manufacturer's instruction.

### Enumeration of multinucleated cells

Phase contrast images were taken using a Zeiss Axiovert 200M microscope. Multinuclear cells were counted from phase contrast images of at least 500 cells per sample. Results from 2 or 3 independent experiments were statistically analyzed using GraphPad Prism software as described in the Figure legends (**Figs. 4 and 5D**; **Fig. S5**). In **Fig. 5E**, to identify Flag-HMG20b expressing cells after transient transfection, cells grown on coverslips were stained with anti-Flag antibody and both phase contrast and fluorescence images were taken using Zeiss Axiovert 200M microscope.

### Immunofluorescence

HeLa Tet-On cell lines containing GFP-HMG20b constructs were grown on glass coverslips and treated with Doxycycline (1 μg/ml) overnight. Cells were fixed in 4% PFA for 5 min, permeabilised with TBS-T (TBS containing 0.1% Triton-X100) and incubated in blocking solution (2% BSA in TBS-T). The cells were then incubated with the indicated primary and secondary antibodies (diluted in blocking solution) for1 hour and 30 minutes respectively. GFP-HMG20b was stained with anti GFP antibody (Living Colors A.v. monoclonal antibody JL-8, Clontech, 632381, 1:100). MKLP1 was stained with anti-MKLP1 rabbit polyclonal antibody (Santa Cruz Biotechnology (N-19, sc-867), 1:1,000)). Anti-HMG20b monoclonal antibody clone 1F6 and rabbit polyclonal antibody against Cep55 were purchased from Abnova (1F6: H00010362-M01, 1:500, Cep55: H00055165-D01, 1:1,000). Tubulin was stained with rat monoclonal anti Tubulin antibody (Abcam, ab6161, 1:500). Rabbit polyclonal anti phospho-Histone H3 (Ser 10) antibody was purchased from Abcam (ab5176, 1:2,500). Secondary antibodies used were Alexa 488 or 568-conjugated goat IgGs from Invitrogen (A11029 and A11036). Images were acquired with Zeiss LSM510 META confocal microscope and analyzed using Image J software.

### Western blotting

Whole-cell extracts were made using NP-40 lysis buffer (50 mM HEPES (pH 7.4), 100 mM NaCl, 0.5% NP-40, 10 mM EDTA, 20 mM β-glycerophosphate, 1mM DTT, 1mM sodium orthovanadate, complete protease inhibitor cocktail (Roche)) and resolved with 4–12% Bis-Tris gel (Invitrogen, NP0335BOX). The antibodies used were anti-HMG20b (mouse monoclonal clone 4.21 (anti-BRAF35, Millipore, 05-641), rabbit polyclonal antibody M10^13^), anti-Flag mouse monoclonal antibody M2 (Sigma, F1804), anti-GAPDH (mouse monoclonal A-3, Santa cruz Biotechnology, sc-137179) and anti-β-Actin (mouse monoclonal AC-15, Sigma, A5441).
