# Supplementary material for: A cancer-associated mutation inactivates a region of the high-mobility group protein HMG20b essential for cytokinesis
Source: Cell Cycle. 2014 Oct 30;13(16):2554–63. doi: 10.4161/15384101.2014.942204 (PMC4614378; doi:10.4161/15384101.2014.942204)
Supplement: 942204_Supplemental_Figures_S1-S4.pdf [file kccy-13-16-942204-s001.pdf]

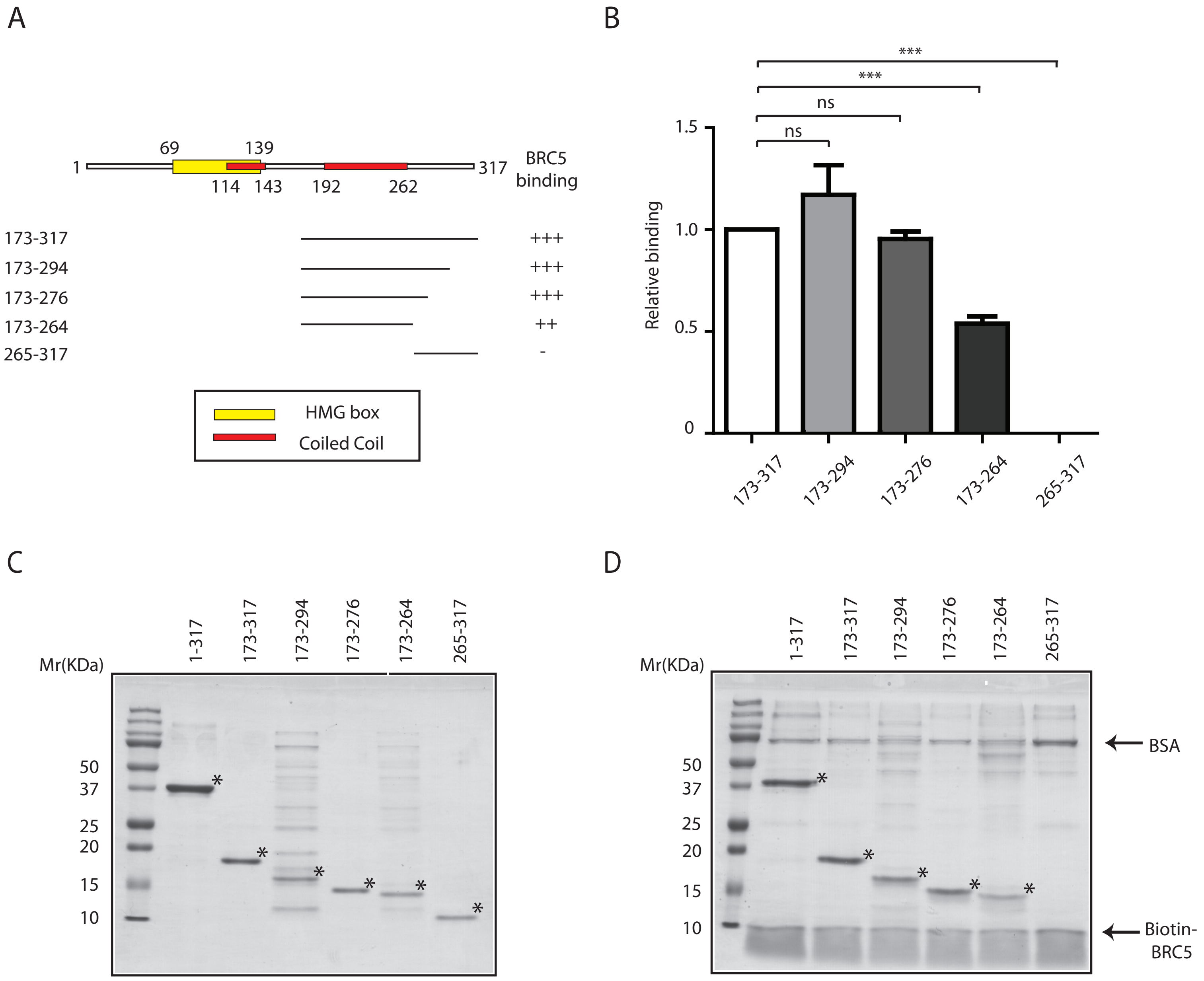

Supplementary Figure S1. Mapping of BRC5 binding region within the C-terminal domain (173-317). (**A**) Design of the truncated C-terminal HMG20b fragments. The boundaries are shown as amino acid residue numbers. Binding affinity for BRC5 is shown next to each fragment. (**B**) Relative binding affinity of C-terminal HMG20b fragment for BRC5 repeat. Binding affinity was calculated by dividing band intensities of bound fractions by those of input. Please note that binding of the 265-317 fragment was undetectable. Relative binding affinity of each fragment compared to the 173-317 fragment is shown as means ± s.e.m. from five independent experiments. Each HMG20b fragment is compared with the 173-317 fragment by Dunnett's multiple comparison test (***: P<0.001, ns: P>0.05). (**C**) Coomassie Brilliant Blue staining of gel showing purified C-terminal HMG20b fragments (marked with an asterisk). (**D**) Coomassie Brilliant Blue staining of gel from Streptavidin pull-down assay with C-terminal fragments. Markings are as shown in Figure 1(**D**).

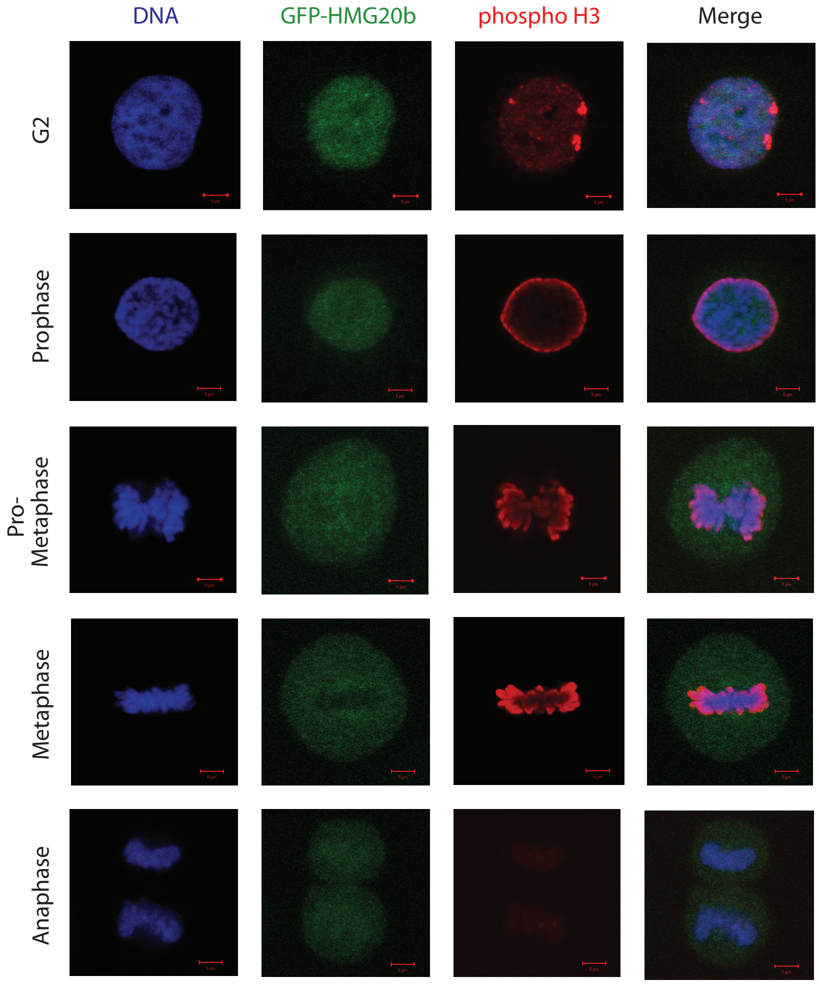

Supplementary Figure S2. HMG20b does not decorate early mitotic chromosomes. HeLa cells expressing GFP-HMG20b (green) were co-stained with anti phospho-Histone H3 (Ser 10) antibody (red) which served as a marker for early mitotic chromosomes. Scale bar is 5μm.

A

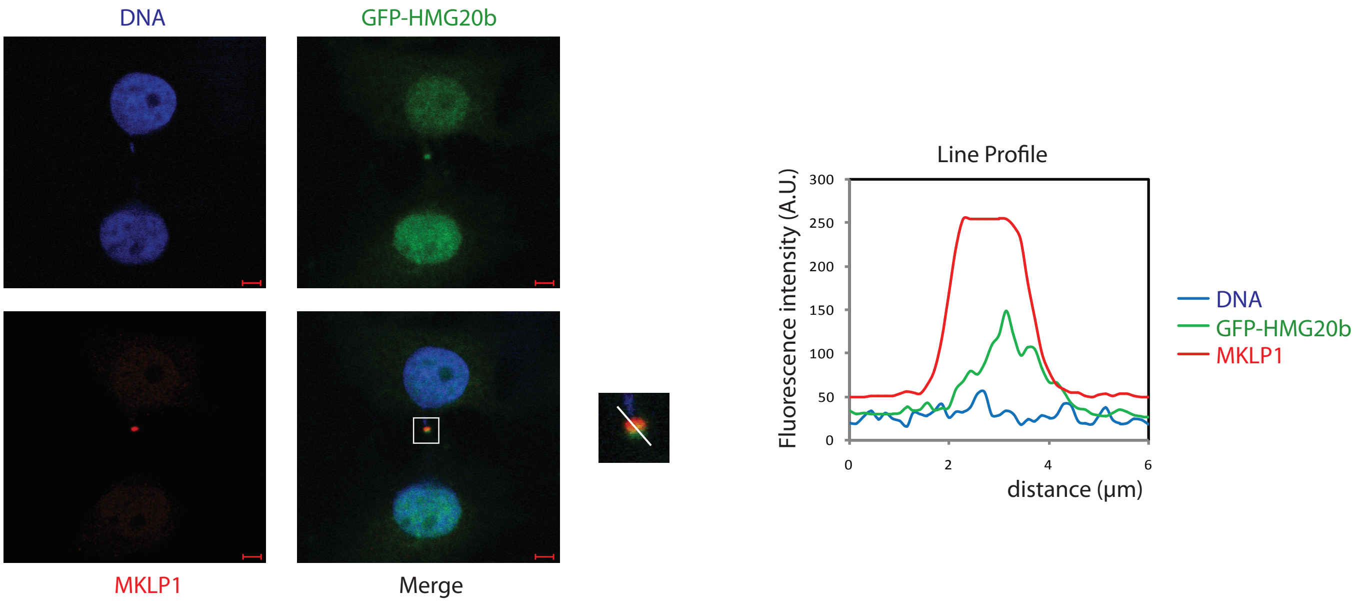

B

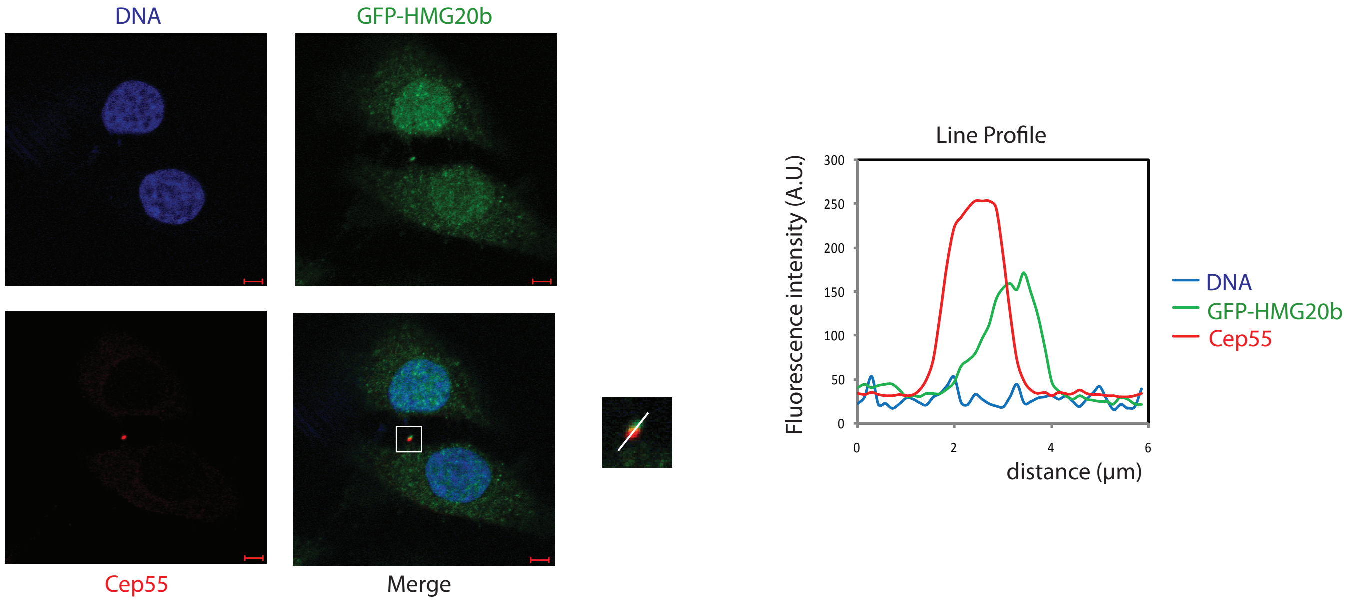

Supplementary Figure S3. Localization of HMG20b at midbody. (**A**) Confocal image of a HeLa cell expressing GFP-HMG20b (green) co-stained with MKLP1 antibody (red). Next to the merged image, an enlarged image of the midbody and a line profile of fluorescence intensity across the midbody are shown. Scale bar is 5μm. (**B**) The same analysis as described in (**A**) with co-staining with Cep55 antibody (red).

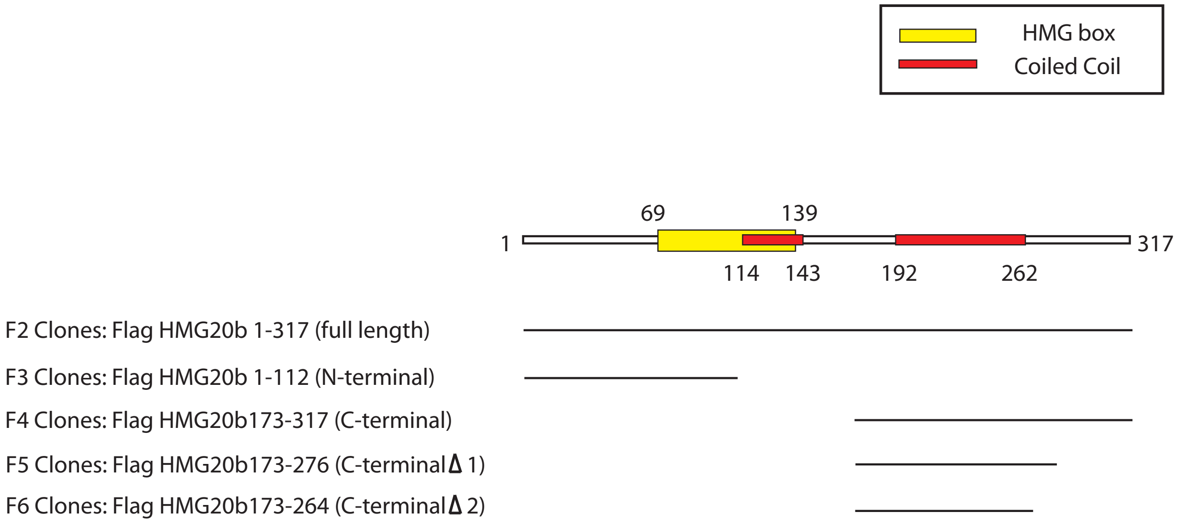

Supplementary Figure S4. A schematic showing the design of Flag HMG20b constructs used to make each series of Tetracycline-inducible cell lines.

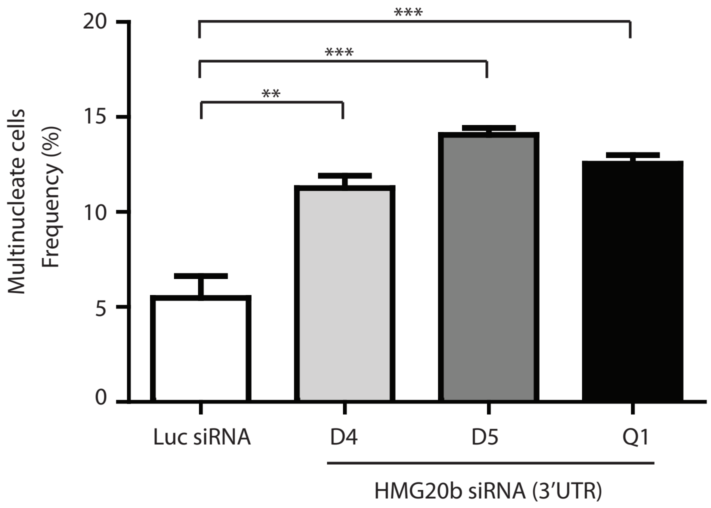

Supplementary Figure S5. Multinucleation frequency measured from phase-contrast images taken 72 hour after transfection of HeLa TetOn cells with control Luciferase (Luc) siRNA and HMG20b siRNAs. Means ± s.e.m. from three independent experiments are shown and >1,000 cells were analyzed per each sample. Each siRNA treatment is compared with the control siRNA by Dunnett's multiple comparison test (***: P<0.001, **: 0.001<P< 0.01).

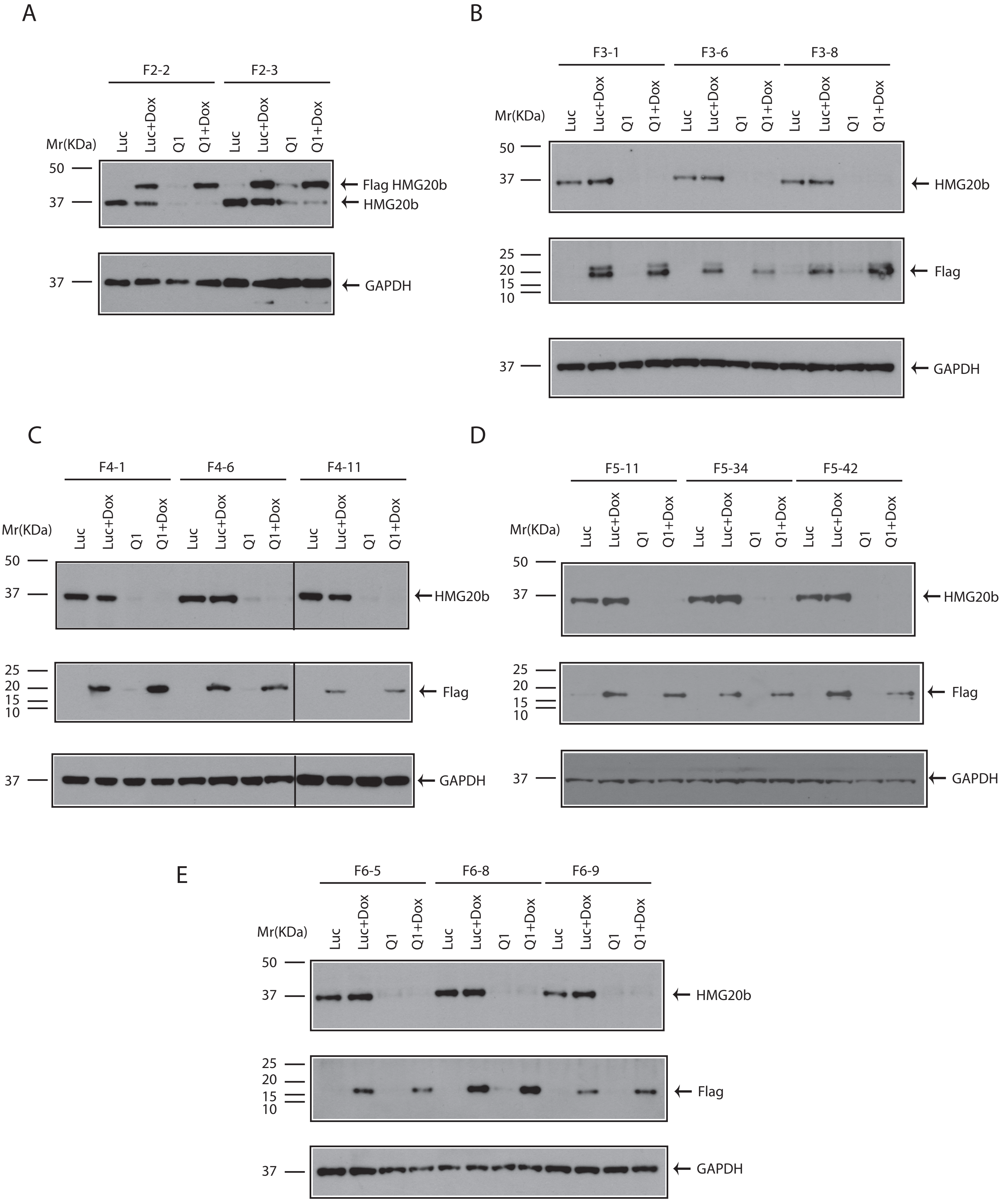
A
F2-2
F2-3
Mr(KDa)
Luc
Luc+Dox
Q1
Q1+Dox
Luc
Luc+Dox
Q1
Q1+Dox
50
37
Flag HMG20b
HMG20b
37
GAPDH
B
F3-1
F3-6
F3-8
Mr(KDa)
Luc
Luc+Dox
Q1
Q1+Dox
Luc
Luc+Dox
Q1
Q1+Dox
Luc
Luc+Dox
Q1
Q1+Dox
50
37
HMG20b
25
20
15
10
Flag
37
GAPDH
C
F4-1
F4-6
F4-11
Mr(KDa)
Luc
Luc+Dox
Q1
Q1+Dox
Luc
Luc+Dox
Q1
Q1+Dox
Luc
Luc+Dox
Q1
Q1+Dox
50
37
HMG20b
25
20
15
10
Flag
37
GAPDH
D
F5-11
F5-34
F5-42
Mr(KDa)
Luc
Luc+Dox
Q1
Q1+Dox
Luc
Luc+Dox
Q1
Q1+Dox
Luc
Luc+Dox
Q1
Q1+Dox
50
37
HMG20b
25
20
15
10
Flag
37
GAPDH
E
F6-5
F6-8
F6-9
Mr(KDa)
Luc
Luc+Dox
Q1
Q1+Dox
Luc
Luc+Dox
Q1
Q1+Dox
Luc
Luc+Dox
Q1
Q1+Dox
50
37
HMG20b
25
20
15
10
Flag
37
GAPDH

Supplementary Figure S6. Tetracycline-inducible expression of HMG20b fragments in HMG20b-depleted cells. (**A**) Two independently isolated clones, F2-2 and F2-3 express Flag-tagged full-length HMG20b following treatment with Doxycycline. At the same time, these clones were transfected with either control (Luciferase) or HMG20b siRNA (Q1) to deplete the endogenous protein. Both exogenous and endogenous proteins were detected with anti HMG20b antibody (Clone 4.21). GAPDH was used as a loading control. (**B**) Clones, F3-1, F3-6, and F3-8 express Flag-tagged N-terminal HMG20b fragment (1-112). (**C**) Clones, F4-1, F4-6, and F4-11 express Flag-tagged C-terminal HMG20b fragment (173-317). The lines between lane 8 and 9 depicts the boundary where two blots were juxtaposed. (**D**) Clones, F5-11, F5-34, and F5-42 express Flag-tagged and truncated C-terminal HMG20b fragment $\Delta 1$ (173-276). (**E**) Clones, F6-5, F6-8, and F6-9 express Flag-tagged and truncated C-terminal HMG20b fragment $\Delta 2$ (173-264). Flag-tagged HMG20b fragments were detected with anti-Flag antibody (**B-E**).

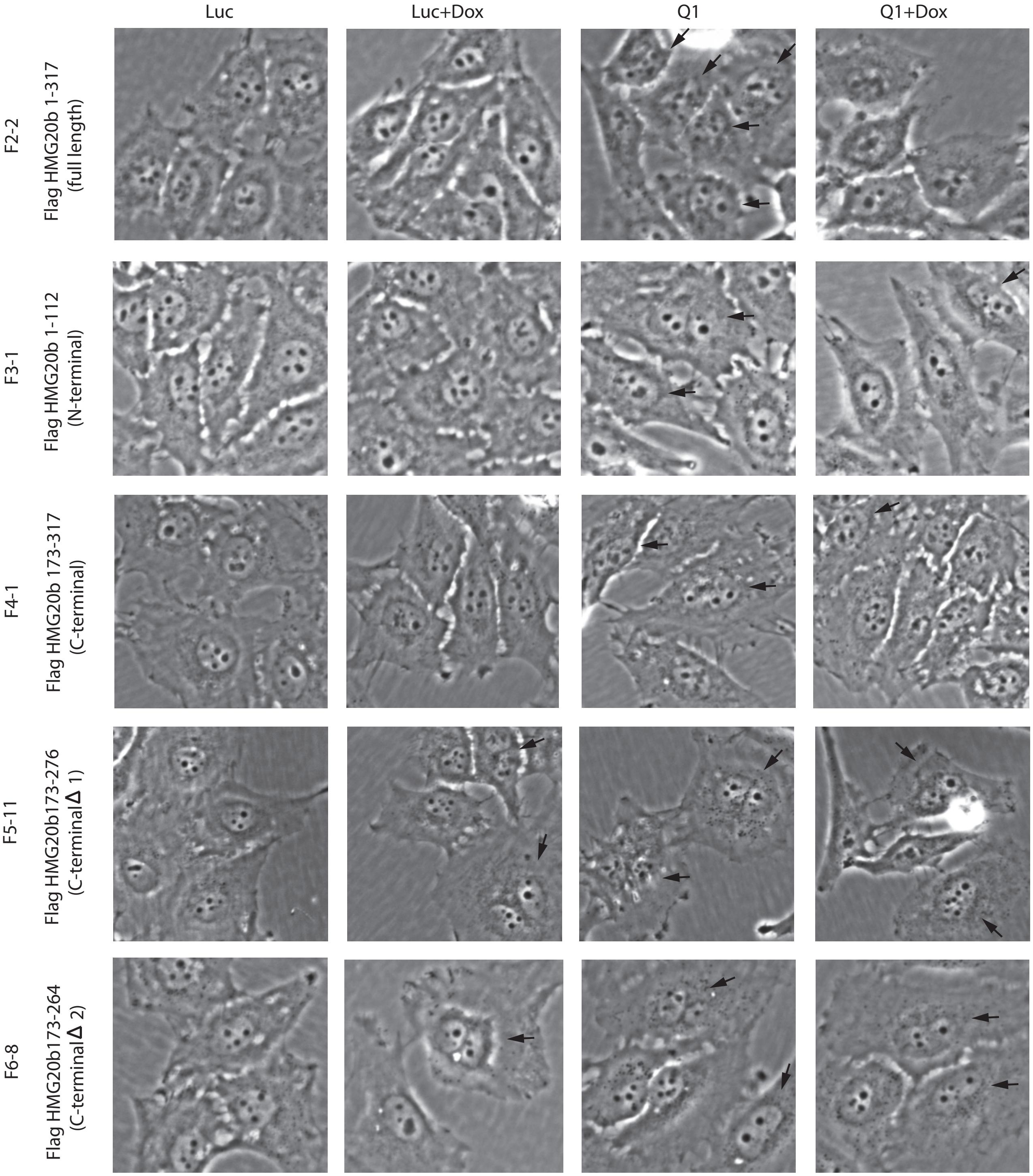

Supplementary Figure S7. Phase-contrast images of inducible HeLa cell lines expressing Flag-HMG20b fragments after treatment with HMG20b siRNA (Q1) and/or Doxycycline (Dox) as described in Figure 4. Representative images from one clone per each fragment are shown. Bi/multinucleated cells are marked with an arrow.

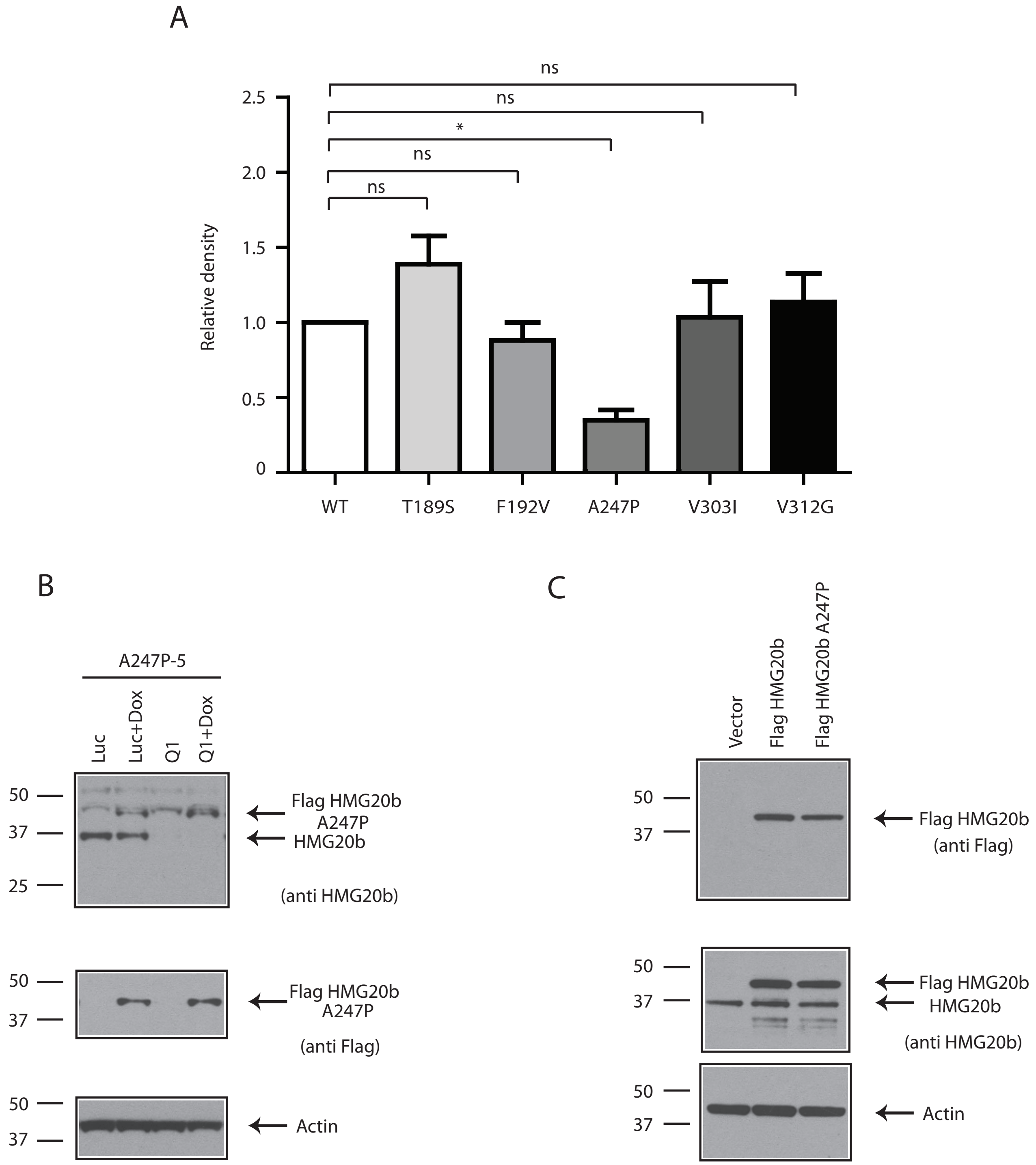

Supplementary Figure S8. Analysis of HMG20b mutants (**A**) Densitometry of GST-pull down assay as shown in Figure 5(**A**). Means ± s.e.m. of relative intensity of Flag-HMG20b bands from three independent experiments are shown. Each mutant is compared with the wild type by Dunnett's multiple comparison test (*: 0.01<P< 0.05, ns: P>0.05). (**B**) Inducible expression of HMG20b A247P mutant in HMG20b-depleted cells (Clone A247P-5). The cells were treated as described in Figure 5(**D**). Actin was used as a loading control. (**C**) Transient expression of Flag-HMG20b wild type and A247P mutant in HeLa cells detected by western blotting using anti-Flag and anti-HMG20b antibodies.

Supplementary Table 1. Cancer-associated mutations in *HMG20B* from COSMIC data base

Position (AA)	CDS Mutation	AA Mutation	Mutation ID (COSM)	Count	Type	Primary Tissue	Histology	Zygosity	Somatic Status
90	c.269C>T	p.T90M	1392426	1	Substitution - Missense	large intestine	carcinoma	Heterozygous	Unknown
103	c.307A>G	p.M103V	1612057	1	Substitution - Missense	liver	carcinoma	Heterozygous	Unknown
114	c.340A>G	p.T114A	1392427	1	Substitution - Missense	large intestine	carcinoma	Heterozygous	Unknown
134	c.402G>A	p.L134L	994911	1	Substitution - coding silent	endometrium	carcinoma	Heterozygous	Unknown
154	c.455_457delAGA	p.K154delK	994912	1	Deletion - In frame	endometrium	carcinoma	Heterozygous	Unknown
189	c.566C>G	p.T189S	1612059	1	Substitution - Missense	liver	carcinoma	Heterozygous	Unknown
192	c.574T>G	p.F192V	994921	1	Substitution - Missense	endometrium	carcinoma	Heterozygous	Unknown
192	c.576C>T	p.F192F	994922	1	Substitution - coding silent	endometrium	carcinoma	Heterozygous	Unknown
247	c.739G>C	p.A247P	1197410	1	Substitution - Missense	lung	carcinoma	Unknown	Unknown
303	c.907G>A	p.V303I	994927	1	Substitution - Missense	endometrium	carcinoma	Heterozygous	Unknown
312	c.935T>G	p.V312G	352041	1	Substitution - Missense	lung	carcinoma	Unknown	
	c.473-1G>C	p.?	459987	1	Unknown	cervix	carcinoma	Unknown	Confirmed Somatic
